# High *p*‐Tau217 Levels Nullify Age‐related Cognitive Protection in Younger Older Adults

**DOI:** 10.1002/alz70856_103605

**Published:** 2025-12-26

**Authors:** Lamia Choity, Xuemei Zeng, Rebecca A Deek, Jeremy M. Gu, Marissa F Farinas, Michel N Nafash, Tara K Lafferty, Margaret A Bedison, Rocco B Mercurio, Cristy Matan, Alexandra Gogola, Julia K. Kofler, Dana L Tudorascu, C. Elizabeth Shaaban, Jennifer H Lingler, Tharick A Pascoal, William E Klunk, Victor L Villemagne, Sarah B Berman, Robert Sweet, Beth E. Snitz, Milos D. Ikonomovic, Ann D Cohen, M. Ilyas Kamboh, Oscar L Lopez, Thomas K Karikari

**Affiliations:** ^1^ University of Pittsburgh School of Medicine, Pittsburgh, PA, USA; ^2^ University of Pittsburgh, Pittsburgh, PA, USA; ^3^ School of Public Health, University of Pittsburgh, Pittsburgh, PA, USA; ^4^ University of Pittsburgh Alzheimer's Disease Research Center (ADRC), Pittsburgh, PA, USA; ^5^ University of Pittsburgh, School of Medicine, Pittsburgh, PA, USA

## Abstract

**Background:**

Plasma *p*‐tau217 is pivotal for diagnosing Alzheimer's Disease (AD) and monitoring its progression, with high *p*‐tau217 levels associated with amyloid pathology and faster cognitive decline. While younger age is generally expected to provide protection against cognitive decline, the interaction between *p*‐tau217 levels and age on the cognitive decline remains poorly understood. Addressing this gap is crucial for improving risk prediction and tailoring interventions across age groups.

**Method:**

Participants at the University of Pittsburgh Alzheimer's Disease Research Center underwent blood collection and Clinical Dementia Rating (CDR) Sum of Boxes assessment cross‐sectionally, followed by annual CDR assessments for up to 29 years (3.0 [IQR 1.9‐5.9]). A subset of participants (*n* = 243) underwent neuroimaging for AD pathologies. Plasma ‐tau217 levels were determined by ALZpath *p*‐tau217 assay. Kaplan‐Meier and Cox proportional hazards models were used to survival analysis, with events defined by an increase in the CDR global score during follow‐up.

**Result:**

A total of 2394 individuals (aged 71.8 ± 9.5 years; 58.3% female; 91.5% self‐identified non‐Hispanic White) were included and divided by age (<70 vs. >=70 years) and *p*‐tau217 levels (threshold of 0.547 pg/ml determined from the sub‐cohort with Aβ PET). Both high *p*‐tau217 and older age were associated with greater hazards of cognitive decline, with hazard ratios of 3.16 (2.82 – 3.53) and 1.67 (1.49 – 1.87), respectively. Age significantly modified the cognitive decline hazard imposed by high *p*‐tau217 (interaction effect *p* < 0.0001). High *p*‐tau217 posed a greater hazard in younger individuals, with a hazard ratio of 9.4 (5.9–15.0) and a median survival time of 3.9 years, compared to 15.8 years for low *p*‐tau217. In older individuals, the hazard ratio was 4.9 (2.9 – 8.5), with a median survival time of 4.0 years compared to 7.9 years for those with low *p*‐tau217. Younger and older individuals with high *p*‐tau217 had similar cognitive decline rates, with median survival times of 4.0 and 3.9 years, respectively (*p* = 0.08).

**Conclusion:**

Our results indicate that high *p*‐tau217 levels negate the protective effect of youth on cognitive decline, highlighting the importance of assessing *p*‐tau217 in younger older adult populations to enable early interventions for Alzheimer's Disease.

